# GITR Activation Positively Regulates Immune Responses against *Toxoplasma gondii*

**DOI:** 10.1371/journal.pone.0152622

**Published:** 2016-03-30

**Authors:** Frederico R. C. Costa, Caroline M. Mota, Fernanda M. Santiago, Murilo V. Silva, Marcela D. Ferreira, Denise M. Fonseca, João S. Silva, José R. Mineo, Tiago W. P. Mineo

**Affiliations:** 1 Laboratório de Imunoparasitologia “Dr. Mário Endsfeldz Camargo”, Instituto de Ciências Biomédicas, Universidade Federal de Uberlândia, Av. Amazonas s/n, Bloco 4C, Sala 4C01, 38405–320, Uberlândia, Minas Gerais, Brazil; 2 Departamento de Bioquímica e Imunologia, Faculdade de Medicina de Ribeirão Preto, Av. dos Bandeirantes 3900, 14049–900, Ribeirão Preto, São Paulo, Brazil; Institut national de la santé et de la recherche médicale—Institut Cochin, FRANCE

## Abstract

*Toxoplasma gondii* is a widespread parasite responsible for causing clinical diseases especially in pregnant and immunosuppressed individuals. Glucocorticoid-induced TNF receptor (GITR), which is also known as TNFRS18 and belongs to the TNF receptor superfamily, is found to be expressed in various cell types of the immune system and provides an important costimulatory signal for T cells and myeloid cells. However, the precise role of this receptor in the context of *T*. *gondii* infection remains elusive. Therefore, the current study investigated the role of GITR activation in the immunoregulation mechanisms induced during the experimental infection of mice with *T*. *gondii*. Our data show that *T*. *gondii* infection slightly upregulates GITR expression in Treg cells and B cells, but the most robust increment in expression was observed in macrophages and dendritic cells. Interestingly, mice infected and treated with an agonistic antibody anti-GITR (DTA-1) presented a robust increase in pro-inflammatory cytokine production at preferential sites of parasite replication, which was associated with the decrease in latent brain parasitism of mice under treatment with DTA-1. Several *in vivo* and *in vitro* analysis were performed to identify the cellular mechanisms involved in GITR activation upon infection, however no clear alterations were detected in the phenotype/function of macrophages, Tregs and B cells under treatment with DTA-1. Therefore, GITR appears as a potential target for intervention during infection by the parasite *Toxoplasma gondii*, even though further studies are still necessary to better characterize the immune response triggered by GITR activation during *T*. *gondii* infection.

## Background

*Toxoplasma gondii* is an ubiquitous protozoan parasite that is estimated to infect one-third of the world’s human population. It can infect many species of warm-blooded animals and is a significant zoonotic and veterinary pathogen [1]. Newly acquired *T*. *gondii* infection in a pregnant woman can be transmitted to the fetus and may cause mental retardation, blindness, epilepsy and death. *T*. *gondii* can also cause severe encephalitis via acute infection or reactivation of latent infections among immunosuppressed individuals, including those with acquired immunodeficiency syndrome, under immunosuppressive cancer therapy, and transplant recipients [2]. *T*. *gondii* has a relatively complex life cycle, with the presence of three main infectious stages: fast replicating tachyzoites, found during acute phases of infection; bradyzoites, which constitute tissue cysts during latent infection; and sporozoites, inside environmental contaminating oocysts [3, 4].

Pathogenicity of *T*. *gondii* is determined by many factors including the susceptibility of the host species, virulence of the parasitic strain, and the infectious stage by which the hosts are exposed. Oocyst-induced infections are most severe in intermediate hosts, and the associated phenomena are not dose-dependent [3]. In order to control the parasite, early production of IL-12 is required [5]. IL-12 commits the adaptive immune response to a Th1-biased profile, causing the lysis of parasites and infected cells by IFN-γ-dependent mechanisms [5, 6]. However, it is known that an exacerbated immune response may lead to undesired inflammatory disorders [6]. In order to preserve tissue integrity, an appropriate immune regulation is required. IL-10 represents one of the major mediators of this regulatory network by controlling both innate and adaptive immune responses [7, 8]. In this context, it has been shown that IL-10 inhibited IL-12, TNF-α and IFN-γ production and prevented the overproduction of T helper 1 type cytokines during *T*. *gondii* infection [5, 8].

GITR, also known as TNFRS18, belongs to the TNF receptor superfamily (TNFRS) which includes CD27, OX40, and 4-1BB [9]. Its signaling provides strong costimulatory signals for T cells when bound to its respective ligand or agonistic antibody (such as the broadly used anti-GITR MAb called DTA-1) [10]. Even though it has been proposed that GITR is a more faithful marker of regulatory T cells [7, 11], GITR is not exclusively expressed in this subset, as observed in experiments using a model of CD25^–^ T cell activation [12]. Of note, other cell types from both hematopoietic and non hematopoietic cell lineages also express GITR at intermediate levels in steady-state, making it difficult to delineate the role of GITR:GITRL interactions *in vivo* [11]. Considering that GITR is widely expressed in different cells of the immune system and that its activation triggers the production of proinflammatory cytokines [13, 14], we evaluated the possible role of ligation-driven GITR activation in the regulation of the immune responses induced by *T*. *gondii* infection.

## Material and Methods

### Ethics Statement

All animal procedures were approved by the institutional ethics committee in animal experimentation (Comissão de Ética no Uso de Animais da Universidade Federal de Uberlândia—Protocol No. 052/12), and were performed based on the Ethical Principles in Animal Research adopted by the Brazilian College of Animal Experimentation.

### Study Design

Spleen cells of naive wild-type C57BL/6 (WT) mice were harvested, rested overnight, and cultured for an additional 24h in the presence of tachyzoites of the ME-49 strain (MOI of 0.1, 0.01 and 0.001), for analysis of the expression of GITR in different cell phenotypes, including CD4^+^FoxP3^-^, CD4^+^FoxP3^+^ and CD8^+^ T cells, B cells, macrophages and dendritic cells by flow cytometry. The effects of GITR ligation by an agonistic antibody anti-GITR (DTA-1) in cytokine production of macrophages were assessed *in vitro*. For the *in vivo* experiments, WT and C57BL/6.Foxp3^GFP/+^ mice were infected through intraperitoneal route with 20 cysts of the ME-49 strain of *T*. *gondii* and sacrificed at different time points in order to compare the effect of the treatment with an agonistic antibody anti-GITR (DTA-1, one day before experimental challenge) with control mice (PBS). Splenocytes from these mice were phenotyped for the percentage of CD4^+^Foxp3^+^, whereas the serum, peritoneal fluid and brain were collected for the measurement of specific antibody titers, cytokine production and parasite burden, respectively. Independent groups and different sets of experiments confirmed the experimental results herein presented.

### Mice

All experiments were carried out with 6–8-week-old wild type C57BL/6 bred and maintained at the animal facilities of the Federal University of Uberlândia (UFU). Foxp3^GFP/+^ C57BL/6 mice were obtained at the animal facilities of the School of Medicine of Ribeirão Preto (USP).

### Parasites

*T*. *gondii* tachyzoites of the ME-49 strain were maintained by serial passages in HeLa cell line (ATCC CCL-2) cultured in RPMI 1640 medium supplemented with 2 mM glutamine, 100 U/mL penicillin, 100 μg/mL streptomycin, and 2% heat-inactivated calf fetal serum (FCS) at 37°C in a 5% CO_2_. For infection experiments, parasites were suspended in RPMI-1640 medium and the number of viable tachyzoites was determined by Trypan blue exclusion in a regular slide hemocytometer. Tissue cysts were obtained from the central nervous system of chronically infected C57BL/6 (30 days). Brains were removed, washed in 0.01 M phosphate-buffered saline (PBS) pH 7.2, homogenized and quantified under light microscopy. For the experimental infection, mice were infected intraperitoneally (i.p.) with 20 cysts per animal.

### DTA-1 monoclonal antibody (MAb)

An hybridoma cell line against GITR (DTA-1, rat IgG2a) was raised following previous description [9]. Briefly, cells were grown in RPMI media, with the addition of 20% fetal bovine serum, and L-glutamine and β-mercaptoethanol, at 37°C in a humidified 5% CO_2_ atmosphere. The supernatants from the cell culture were collected, centrifuged at 700 x g, for 10 minutes, at 4°C. Pellet was discarded, and the supernatant was concentrated in saccharose and again centrifuged. The supernatant was then purified by gel and affinity filtration by Sephacryl G-300 and Protein G columns, respectively. Protein was quantified by the Bradford method.

### In vitro culture of bone marrow-derived macrophages

Immortalized bone marrow-derived macrophages (BMDMs) were obtained as described [15]. The cells were counted in an haemocytometer and plated (2x10^5^/well) in 96-well plates and incubated at 37°C, with 5% CO_2_. After 18h hours, these cells were stimulated or not with DTA-1 (anti-GITR antibody, 100mg/ml) or Rat serum (irrelevant antibody, 100mg/ml) and infected with different rates of tachyzoites of the ME-49 strain of *T*. *gondii* tachyzoites, for eighteen hours. The culture supernatant was stored at -80°C for cytokine determination by ELISA, according to the manufacturer’s recommendation.

### Cell phenotyping and GITR expression

Spleen cells from WT mice were co-cultured with live ME-49 tachyzoites (MOIs: 0.1, 0.01 and 0.001). After 24 hours, cell suspensions were incubated with PBS plus 10% rabbit serum to block nonspecific binding of the antibodies, and labeled with fluorochrome-conjugated antibodies against (all from BD Biosciences, San Diego, USA): CD3 (PerCp), CD4 (APC), CD8α (APC-Cy7), CD19 (PE-Cy7), CD11b (APC-Cy7), CD11c (Pe-Cy7), F4/80 (PerCP) and GITR (PE). In an additional experiment, spleen cells from Foxp3^GFP/+^ C57BL/6 mice infected with ME-49 (20 cysts/mouse), submitted or not to a single dose of DTA-1 (500 μg/mouse/one day before experimental infection), were obtained after 5 dpi and phenotyped for T CD4^+^Foxp3^+^ expression. Also, naïve and infected BMDMs were stained for CD11b (APC-Cy7) and GITR (PE) after 24 h. Cell suspensions were read by flow cytometry (FACSCanto II, BD Biosciences), and data analysis was performed using FlowJo (TreeStar Inc., Ashland, OR, EUA).

### Cytokine production

In order to determine the cytokine profile of WT mice infected with ME-49 strain (20 cysts/mouse) and submitted or not to DTA-1 MAb treatment (500 μg/mouse), peritoneal fluids and lungs were collected after 5 dpi and promptly quantified for IFN-γ, TNF-α, IL-6 and IL-10 levels by ELISA or using a mouse Th1/Th2/Th17 cytometric bead array kit (CBA), according to the manufacturer’s instructions (BD Biosciences). Supernatants from in vitro assays with BMDMs were quantified for IL-12p40 and IL-10. The ELISA assays were read at 450 nm and the OD values obtained were converted to pg/mL by the extrapolation of the standard curve (M2e plate reader, Molecular Devices, Sunnyvale, CA, USA); whereas CBA analysis were read in a flow cytometer (FACSCanto II, BD Biosciences), and the concentration of each analyte was extrapolated from standard curves of recombinant cytokines.

### Determination of parasite burden

C57BL/6 mice were treated with DTA-1 (500 μg/mouse; single dose—24h before; or two doses—24h before and 7 days after infection) and infected with 20 cysts per mouse, by intraperitoneal route. Each group was accompanied by its control groups, composed of untreated and infected mice [16]. After 30 days of the infection, mice were sacrificed and had their brain collected for parasitism determination. The number of cysts was determined by light microscopy [17].

### Determination of *T*. *gondii*-specific antibodies

Levels of *T*. *gondii*-specific total IgG, IgG1 and IgG2a antibodies were measured by ELISA as described elsewhere [18], with modifications. Samples were collected at 10, 20 and 30 dpi, and the sera stored at −20°C for further analysis. High-affinity microtiter plates were coated with soluble tachyzoite antigens (STAg, 10 μg/ml), washed with PBS plus 0.05% Tween 20 (PBS-T) and blocked with 5% skim milk in PBS-T for 1 h at room temperature. Serum samples were diluted 1:25 in 1% skim milk-PBS-T and incubated for 1 h (for IgG detection) or 2 h (for IgG1 and IgG2a detection) at 37°C. After washing, peroxidase-labeled goat anti-mouse IgG (1:1000; Sigma Chemical Co., St Louis, MO) or biotin-labeled goat anti-mouse IgG1 (1:4000) or anti-mouse IgG2a (1:2000) antibodies (Caltag Lab. Inc., South San Francisco, CA) were added and incubated for 1 h at 37°C. Next, streptavidin-peroxidase (1:1000; Sigma) was added for IgG1 and IgG2a detection assays. The assays were developed with 0.01 M 2,2-azino-bis-3-ethyl-benzthiazoline sulfonic acid (ABTS; Sigma) and 0.03% H_2_O_2_. Optical density (OD) values were determined in a plate reader at 405 nm. Results were expressed in ELISA index (EI), according to the formula: EI = OD sample/OD cut off, where cut off was calculated as the mean OD for negative control sera plus three standard deviations.

### Statistical analysis

Results were expressed as mean ± SEM. Statistical analysis was performed using the software GraphPad Prism version 5.0 for Windows (GraphPad Software, EUA) using analysis of variance (ANOVA) followed by the Tukey test. Values of P < 0.05 were considered significant.

## Results

### GITR expression is increased in immune cells exposed to *T*. *gondii* tachyzoites

**Fig 1:**
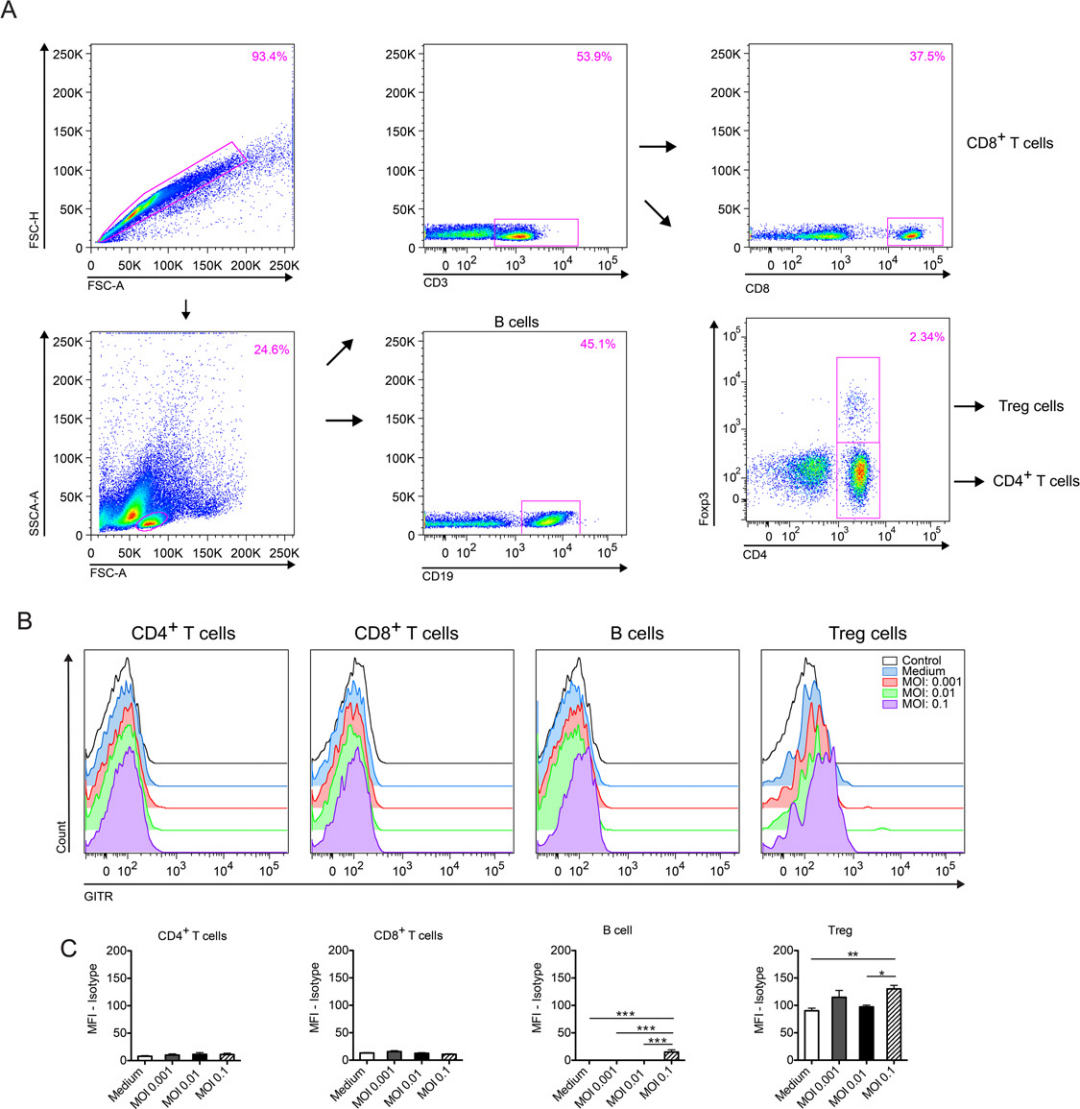
GITR expression in the lymphocyte compartment. Splenocytes from naïve C57BL/6 mice were collected, stimulated or not with tachyzoites of the ME-49 strain at different MOIs (MOI: 0.1; 0.01 and 0.001) and kept in culture for 24 hours. GITR expression was then analyzed by flow cytometry. Gating strategy is depicted in (A), histogram plots in (B) and MFI expression in (C). The lymphocytes were first identified and gated based on their FSC and SSC profile after doublets exclusion. Treg cells were considered as CD3^+^CD4^+^Foxp3^+^ T cells gated on the lymphocyte region. Error bars indicate mean +/- SEM. *P < 0.05 and **P < 0.01. The experiment was performed twice with six technical replicates in each experiment.

**Fig 2:**
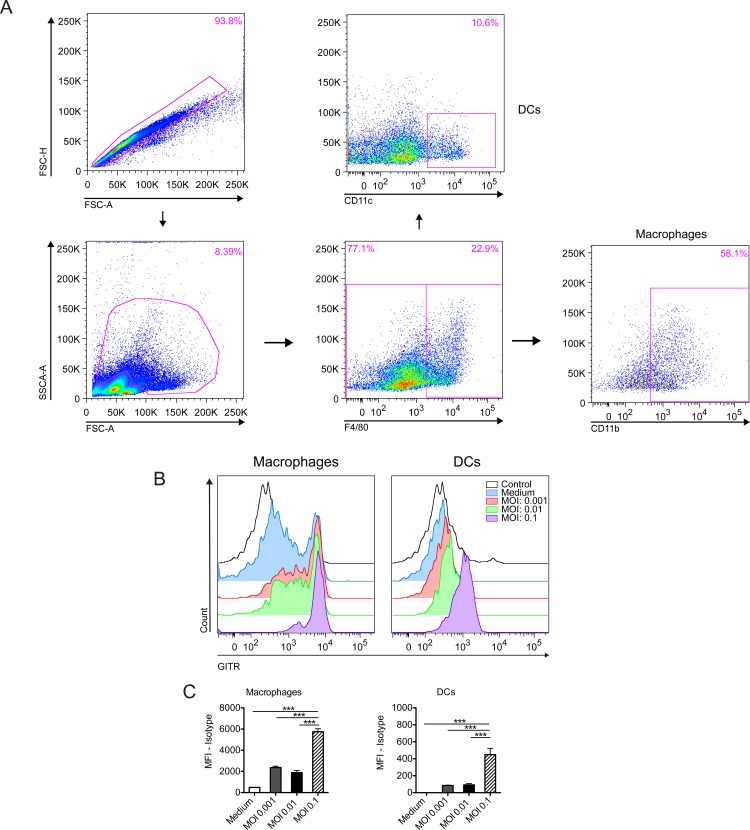
GITR expression in the myeloid compartment. Splenocytes from naïve C57BL/6 mice were collected, stimulated or not with tachyzoites of the ME-49 strain at different MOIs (MOI: 0.1; 0.01 and 0.001) and kept in culture for 24 hours. GITR expression was then analyzed by flow cytometry. Gating strategy is depicted in (A), histogram plots in (B) and MFI expression in (C). The myeloid cells were first identified and gated based on their FSC and SSC profile after doublets exclusion. Macrophages were considered as F4/80^+^CD11b^+^ and dendritic cells as F4/80^-^CD11c^+^ gated on the myeloid cell region. Error bars indicate mean ± SEM. ***P < 0.001. The experiment was performed twice with six technical replicates in each experiment.

Since different cells of the immune system may express GITR, changes in receptor expression were evaluated in both innate and adaptive immune cells in the context of *T*. *gondii* infection *in vitro*. For that, spleen cells from naïve WT C57BL/6 mice were co-cultured with *T*. *gondii* tachyzoites using different MOIs (0.1, 0.01 and 0.001) for 24h and analyzed by flow cytometry. The starting point at MOI of 0.1 was standardized in-house, in order to reflect a more physiological condition, since macrophage/dendritic cell populations represent between 10–20% of the immune cells contained within the spleen of a mouse. Gating strategy is depicted in Figs 1A and 2A. We observed a slight increase in GITR expression in Treg cells (CD3^+^CD4^+^Foxp3^+^—Fig 1B and 1C) and B cells (CD19^+^—Fig 1B and 1C). Surprisingly, we detected a robust increase in GITR expression after stimulation with ME-49 tachyzoites in macrophages (F4/80^+^CD11b^+^—Fig 2B and 2C) and, in a lesser extent, dendritic cells (F4/80^-^CD11c^+^—Fig 2B and 2C). GITR expression in NK and NKT cells remained unaltered after stimulation (data not shown). Collectively, these results suggest that GITR signaling may affect multiple cell lines during immune responses mounted against *T*. *gondii*.

### GITR activation potentiates cellular immune responses and reduces chronic phase parasite burden during *in vivo T*. *gondii* infection

**Fig 3:**
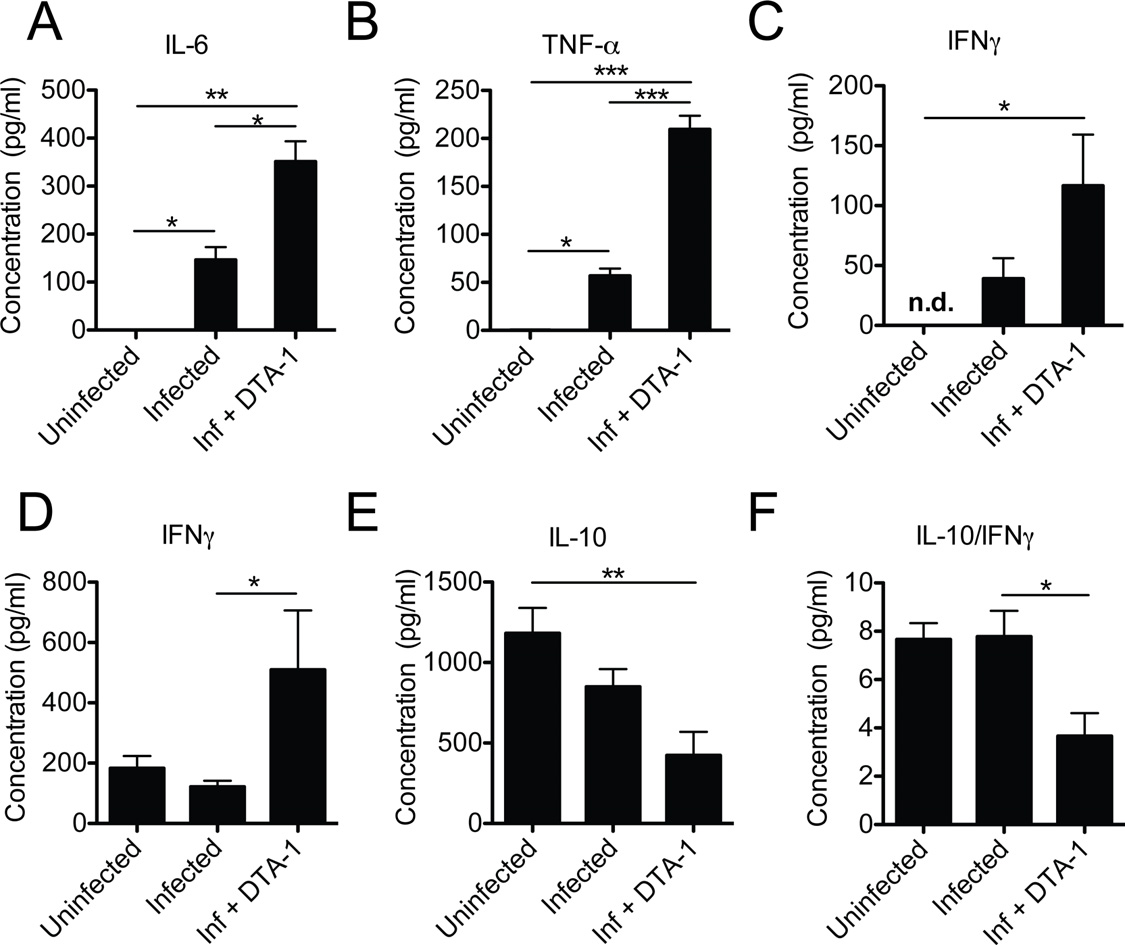
Cytokine production is largely altered by DTA-1 in response to *T*. *gondii* infection *in vivo*. (A, B and C) C57BL/6 mice were treated or not with 500 μg of DTA-1 one day before experimental infection. In the control group PBS was administered. Infection was performed with 20 cysts injected i.p. Five days later mice were euthanized and the peritoneal fluid was collected and promptly analyzed by CBA. (D) C57BL/6 mice infected with ME-49 submitted or not to a single dose treatment with DTA-1 were sacrificed five days after infection and had their lungs collected for the cytokine expression analysis by ELISA. Results were expressed as mean +/- SEM. *P<0.05, **P<0.01 and ***P<0.001 (n = 5/group).

To further evaluate the role of GITR activation upon *T*. *gondii* infection *in vivo*, we analyzed the production of multiple cytokines (Fig 3A–3C) in the peritoneal fluid (5 dpi) of mice either infected and treated with DTA-1 or infected alone. As expected, mice treated with DTA-1 MAb produced higher levels of the inflammatory cytokines IL-6 (Fig 3A), TNF-α (Fig 3B) and IFN-γ, though the latter did not reach statistical difference compared to infected mice alone (Fig 3C).

We next addressed whether this cytokine balance was reflected in tissues affected by acute phase parasite replication, such as the lungs [19]. In order to test this hypothesis, WT mice infected with *T*. *gondii*, treated or not with a single dose of DTA-1 were sacrificed 5 days after infection and had their lungs homogenized and analyzed for IFN-γ and IL-10 production. Interestingly, there was a robust increase in IFN-γ production in the lungs of mice treated with DTA-1, whereas the IL-10 production decreased if compared to untreated controls (Fig 3D and 3E, respectively). Even though there was no statistical difference in IL-10 production between DTA-1 –treated and mice infected alone (Fig 3E), *in vivo* GITR activation with DTA-1 decreased the IL-10/IFN-γ ratio in the lungs (Fig 3F). Collectively, these results indicate that GITR ligation stimulates the production of pro-inflammatory mediators systemically and at sites of parasite replication.

**Fig 4:**
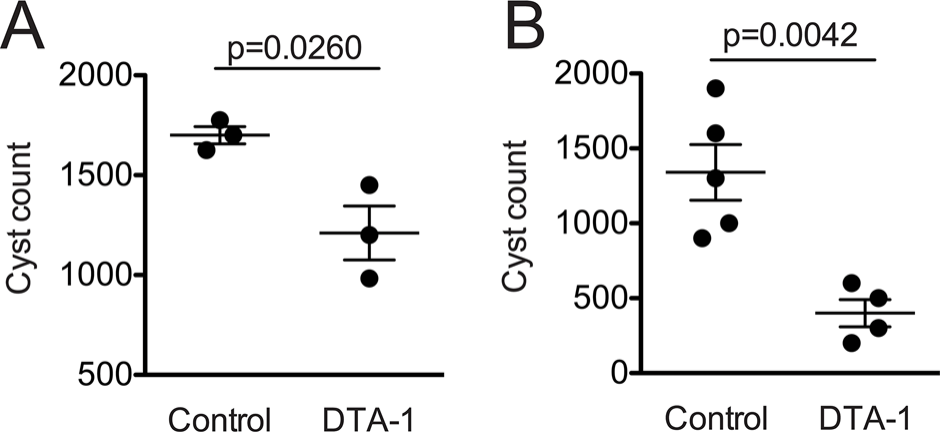
Parasite burden in the chronic phase of the disease is reduced by the treatment with DTA-1. C57BL/6 mice were treated with 500 μg of DTA-1 on days (A) -1 (single dose) and (B) 7 (two doses) after experimental infection. In the control group PBS was administered. Infection was performed with 20 cysts injected i.p. Thirty days later, mice were sacrificed and had their brain collected for parasitism analysis under a light microscope. Results were expressed as mean +/- SEM (n = 5/group).

Since we observed a robust pro-inflammatory immune response in *T*. *gondii*-infected mice treated with DTA-1, we then analyzed the chronic phase parasitism in order to evaluate if GITR activation could influence parasite latency and consequent host resistance. In order to verify that, brain parasitism in WT mice infected with ME-49 and treated with DTA-1 was assessed in the beginning of the chronic stage (30 dpi–Fig 4A and 4B, respectively). There was a significant decrease of brain parasitism after DTA-1 treatment with either one or two doses administered. When mice were treated with a single dose, there was a 29% decrease in the parasitism (mean cyst numbers decreased from 1700 to 1211 after treatment). With two doses, the reduction was even higher (70%), as the mean of cyst numbers dropped from 1340 to 400 after DTA-1 treatment. These data indicate that GITR activation contributes to *T*. *gondii* clearance and consequent host resistance.

### Cellular mechanisms involved in GITR activation is still undetermined

**Fig 5:**
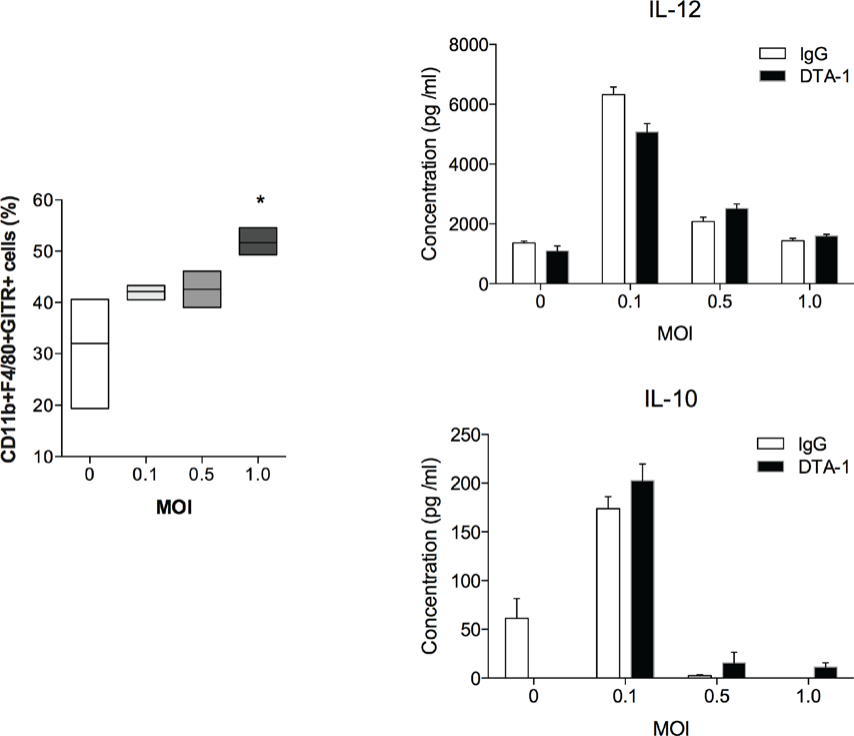
GITR activation does not affect cytokine production by macrophages. Immortalized bone marrow derived macrophages (iBMDM) were plated (2x10^5^/well) and stimulated or not with DTA-1 (anti-GITR antibody, 100 μg/ml) or Rat serum (irrelevant antibody, 100 μg/ml) and infected with different rates of tachyzoites of the ME-49 strain of *T*. *gondii* tachyzoites, for eighteen hours. The cells were washed and assessed for the expression of GITR by flow cytometry (A). Results were shown as the percentage of GITR^+^ cells gated on CD11b^+^F4/80^+^ cells. Alternatively, the culture supernatant was collected and analyzed for IL-12p40 (B) and IL-10 (C) by ELISA. Error bars indicate mean +/- SD. *P < 0.05.

Considering the changes observed in GITR expression, we then tried to identify which cells from the immune system could be responsible for the phenomena observed *in vivo* upon receptor engagement by the monoclonal antibody. First, we assessed macrophages, which had the most pronounced alteration in GITR expression after *in vitro* infection with *T*. *gondii* (Fig 2C). In agreement with the previous assays performed with spleen macrophages, an increment in immortalized bone marrow derived macrophages (iBMDM) GITR expression was observed in an infectious dose-dependent manner (Fig 5A). However, macrophages co-cultured with or without DTA-1 produced similar amounts of IL-12p40 and IL-10 against different MOI of *T*. *gondii* tachyzoites (Fig 5B), ruling out its probable role in enhanced immunity against the infection upon *in vivo* activation of GITR.

**Fig 6:**
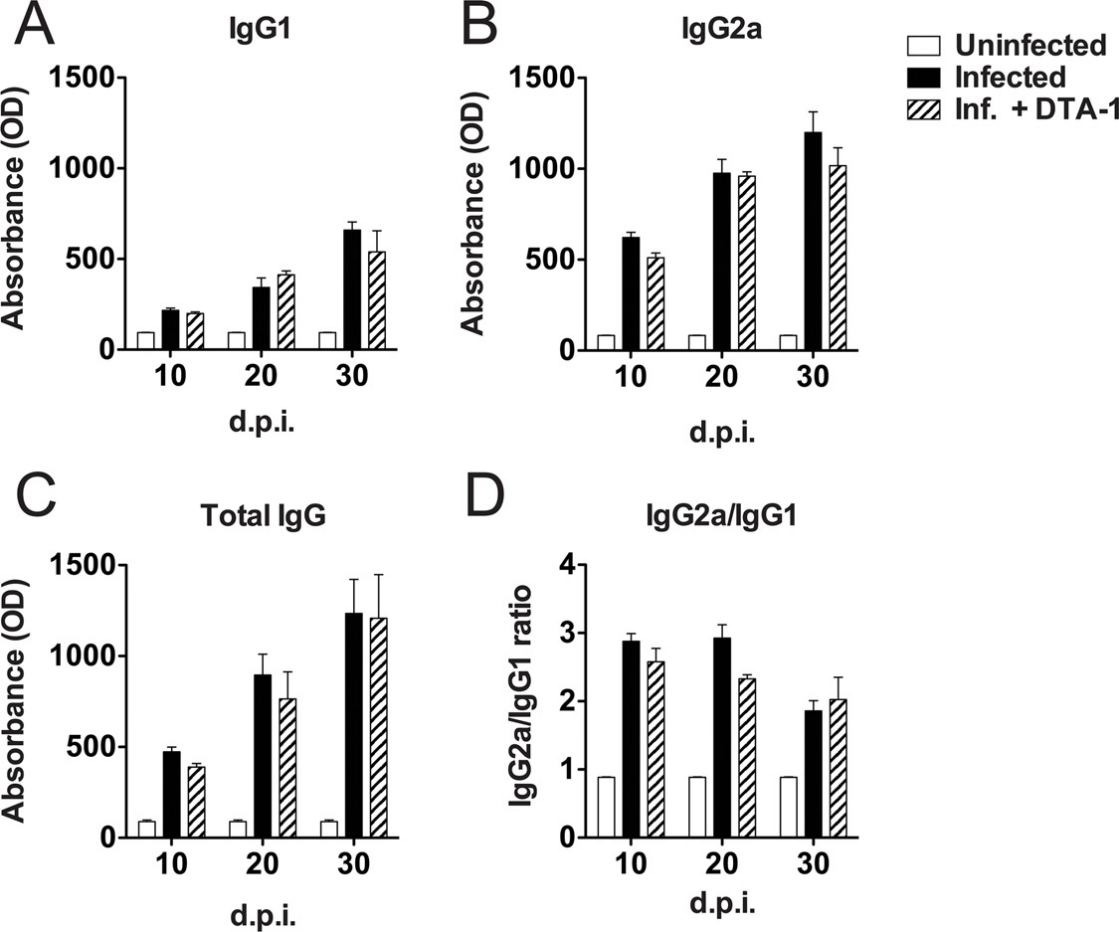
GITR activation does not alter antibody production. C57BL/6 mice were treated or not with 500 μg of DTA-1 one day before experimental infection. In the control group, PBS was administered. Infection was performed with 20 cysts injected i.p. A blood sample was collected on days 10, 20 and 30 after infection. Levels of IgG1 (A), IgG2a (B), Total IgG (C) and IgG2a/IgG1 ratio (D) were assessed by ELISA. Error bars indicate mean +/- SEM (n = 5/group).

**Fig 7:**
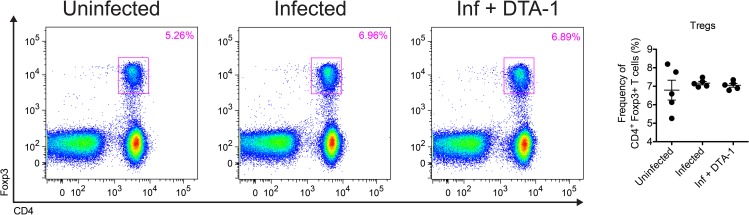
GITR activation has no effect in the Treg cell compartment during *T*. *gondii* infection. C57BL/6 Foxp3^GFP/+^ mice were treated or not with one dose of DTA-1 (500 μg/mouse) one day before the infection with 20 cysts of *T*. *gondii* i.p. Mice were euthanized at 5 dpi and had their spleens collected and analyzed for Foxp3 expression by flow cytometry. Results were shown as the percentage of CD4^+^Foxp3^+^ cells gated on CD3^+^ cells (300,000 events). Each FACS plot corresponds to a representative individual mouse. Error bars indicate mean +/- SEM. The experiment was performed twice, with n = 5 mice/group.

Although GITR expression was slightly modified in the presence of *T*. *gondii tachyzoites*, we also assessed whether DTA-1 treatment could affect specific antibody production in B cells and the number of Tregs during infection. In that sense, we measured the production kinetics of specific IgG antibodies, along with its subclasses IgG1 and IgG2a, in the serum of mice infected with *T*. *gondii*, treated or not with DTA-1. This assay showed that specific IgG production to parasite soluble antigens remained unchanged after treatment with DTA-1 (Fig 6), indicating that increased GITR signaling due to agonist antibody treatment did not alter antibody production and subclass profile in the course of *T*. *gondii* infection. In addition, previous reports using other experimental models have highlighted the role of GITR expression in Treg cells [20], so we evaluated whether GITR activation would affect the proportion of Tregs during infection in FoxP3^GFP/+^ mice. As shown in Fig 7, the activation of GITR with DTA-1 did not result in significant differences in T CD4^+^FoxP3^+^ cells after 5 days of *T*. *gondii* infection *in vivo*. Collectively, these results indicate that GITR activation is not involved in cytokine production by macrophages, antibody production by B cells or in proliferation by Treg cells.

## Discussion

The main goal of this study was to evaluate the possible role of GITR activation during the infection by the parasite *T*. *gondii* in mice. Until now, there is no description in the literature regarding this subject. Our data show changes in GITR expression following *T*. *gondii* infection in Treg cells, B cells, macrophages and dendritic cells, which supports the idea that the GITR signaling pathway might be triggered during infection. To further evaluate whether the activation of GITR signaling would have any effect in the outcome of infection, we triggered its activation using the agonistic antibody DTA-1. Surprisingly, there was a robust increase in IFN-γ production alongside a decrease in the IL-10/IFN-γ ratio in the lungs of infected mice that were treated with DTA-1. Interestingly, GITR activation with DTA-1 also led to an increase in the levels of other pro-inflammatory cytokines, such as IL-6 and TNF-α in the peritoneal cavity. Next, we assessed whether all these alterations in the immune response: robust increase in GITR expression in several cells from the immune system, together with the increase in IFN-γ, TNF-α, and IL-6 production and a decreased IL-10/ IFN-γ at sites of parasite replication in the acute stage of infection, would lead to an increased parasite killing in the chronic stage of the disease. Interestingly, we observed a decrease in brain parasitism in the chronic stage, what could indicate that all these alterations in the acute stage, leading to a more pro-inflammatory profile, could be related to a more efficient parasite killing in an early phase of the disease, making it less likely for high numbers of parasites to reach the brain during the chronic phase of the infection.

Our next step was to identify which cells were responsible for this shift toward a pro-inflammatory microenvironment *in vivo* after GITR engagement with DTA-1. The majority of the recent studies on GITR focus on regulatory T cells, since the common sense in the scientific community is that GITR is highly expressed in these cells, even when they are not activated or stimulated [7, 20]. Dendritic cells, macrophages and B cells are reported to express low levels of the molecule, therefore studies of GITR on these populations are rarely found [20]. Our results partly corroborate with those findings, since GITR expression in Treg cells is increased in steady-state condition if compared to B cells, CD4^+^FoxP3^-^ and CD8^+^ T cells. However, our data do not indicate that GITR plays a significant role in regulatory T cell proliferation during infection by *T*. *gondii*, since DTA-1 treatment did not alter the Treg cell compartment after infection. Whether GITR engagement by DTA-1 affects the suppressive function of Tregs or their cytokine production still remains to be determined.

Considering that we found a robust increase in GITR expression especially in macrophages, we analyzed whether GITR engagement would have any effect on the cytokine milieu induced by *T*. *gondii* in this cell line. Interestingly, no differences in cytokine production *in vitro* by *T*.*gondii*-infected iBMDM stimulated with DTA-1 were found. Thus, GITR engagement *in vitro* with DTA-1 does not play a role in cytokine production by macrophages. However, further studies are still needed to assess whether GITR engagement affects the proliferation, opsonization or the phagocytosis status of macrophages.

Lastly, to rule out a possible phenotype in B cells, which also slightly increased GITR expression upon contact with *T*. *gondii* (MOI 0.1), we assessed whether DTA-1 treatment would alter antigen-specific antibody production in the course of the infection. However, no differences were observed between those groups, indicating that GITR activation is dispensable for antibody production in *T*. *gondii* infection. Since GITR is dispensable for B cell development [21] and antibody production, whether GITR plays a role in antigen presentation by B cells remains to be determined.

In conclusion, our results indicate that GITR engagement with the MAb anti-GITR, DTA-1 induces a robust pro-inflammatory immune response and consequent parasite killing, thus decreasing latency of the parasite in the brain. Also, GITR engagement does not affect cytokine production by macrophages, antibody production by B cells or proliferation of Treg cells. Hence, further studies are necessary to evaluate the molecular mechanisms involved in GITR activation, which is a molecule that emerges as a potential target for intervention in the context of *T*. *gondii* infection.

## Abbreviations

GITR: glucocorticoid-induced TNF receptor
Treg: regulatory T cells
MAb: monoclonal antibody
